# Vaccine‐breakthrough infection by the SARS‐CoV‐2 omicron variant elicits broadly cross‐reactive immune responses

**DOI:** 10.1002/ctm2.720

**Published:** 2022-01-26

**Authors:** Runhong Zhou, Kelvin Kai‐Wang To, Qiaoli Peng, Jacky Man‐Chun Chan, Haode Huang, Dawei Yang, Bosco Hoi‐Shiu Lam, Vivien Wai‐Man Chuang, Jian‐Piao Cai, Na Liu, Ka‐Kit Au, Owen Tak‐Yin Tsang, Kwok‐Yung Yuen, Zhiwei Chen

**Affiliations:** ^1^ AIDS Institute, Li Ka Shing Faculty of Medicine University of Hong Kong Pokfulam Hong Kong Special Administrative Region People's Republic of China; ^2^ Department of Microbiology Li Ka Shing Faculty of Medicine University of Hong Kong Pokfulam Hong Kong Special Administrative Region People's Republic of China; ^3^ State Key Laboratory for Emerging Infectious Diseases University of Hong Kong Pokfulam Hong Kong Special Administrative Region People's Republic of China; ^4^ Centre for Virology, Vaccinology and Therapeutics Limited University of Hong Kong Hong Kong Special Administrative Region People's Republic of China; ^5^ Department of Clinical Microbiology and Infection Control University of Hong Kong‐Shenzhen Hospital Shenzhen Guangdong People's Republic of China; ^6^ National Clinical Research Center for Infectious Diseases The Third People's Hospital of Shenzhen and The Second Affiliated Hospital of Southern University of Science and Technology Shenzhen Guangdong People's Republic of China; ^7^ Department of Medicine and Geriatrics Princess Margaret Hospital Hong Kong Special Administrative Region People's Republic of China; ^8^ Department of Pathology Princess Margaret Hospital Princess Margaret Hospital Hong Kong Special Administrative Region People's Republic of China; ^9^ Quality & Safety Division Hospital Authority Hong Kong Special Administrative Region People's Republic of China

To the Editor:

After the World Health Organization (WHO) designated the omicron variant of concern (VOC) on the 26 November 2021, the extremely rapid spread of this variant is replacing the delta VOC with increased risk of vaccine‐breakthrough infection in the South Africa, in European countries and in the United States.^1^ Both vaccine‐induced neutralising antibodies (NAbs) and current NAbs in combination therapy have shown significantly reduced activities.^2^, ^3^ Till now, it remains unclear whether vaccine‐induced memory responses can be recalled by the omicron viral infection. We, therefore, investigated the host immune responses in two cases of vaccine‐breakthrough omicron infection in Hong Kong.

In mid‐November 2021, the first Chinese case of omicron patient (OP1) was diagnosed in a quarantine hotel in Hong Kong.^4^ About 9 days after the OP1, omicron patient 2 (OP2), who was due to a separate transmission event, was also confirmed by sequencing analysis. Based on the vaccination records, OP1 and OP2 were confirmed with omicron infection at 178 and 53 days after the second dose of BNT162b2 and mRNA‐1273, respectively (Figure 1A). During hospitalisation, both cases presented with mild clinical symptoms not requiring oxygen supplementation or ICU treatment. With patients’ informed consent, we obtained three sequential sera and one peripheral blood mononuclear cell (PBMC) samples from each patient to determine their immune responses recalled by the omicron viral infection.

**FIGURE 1:**
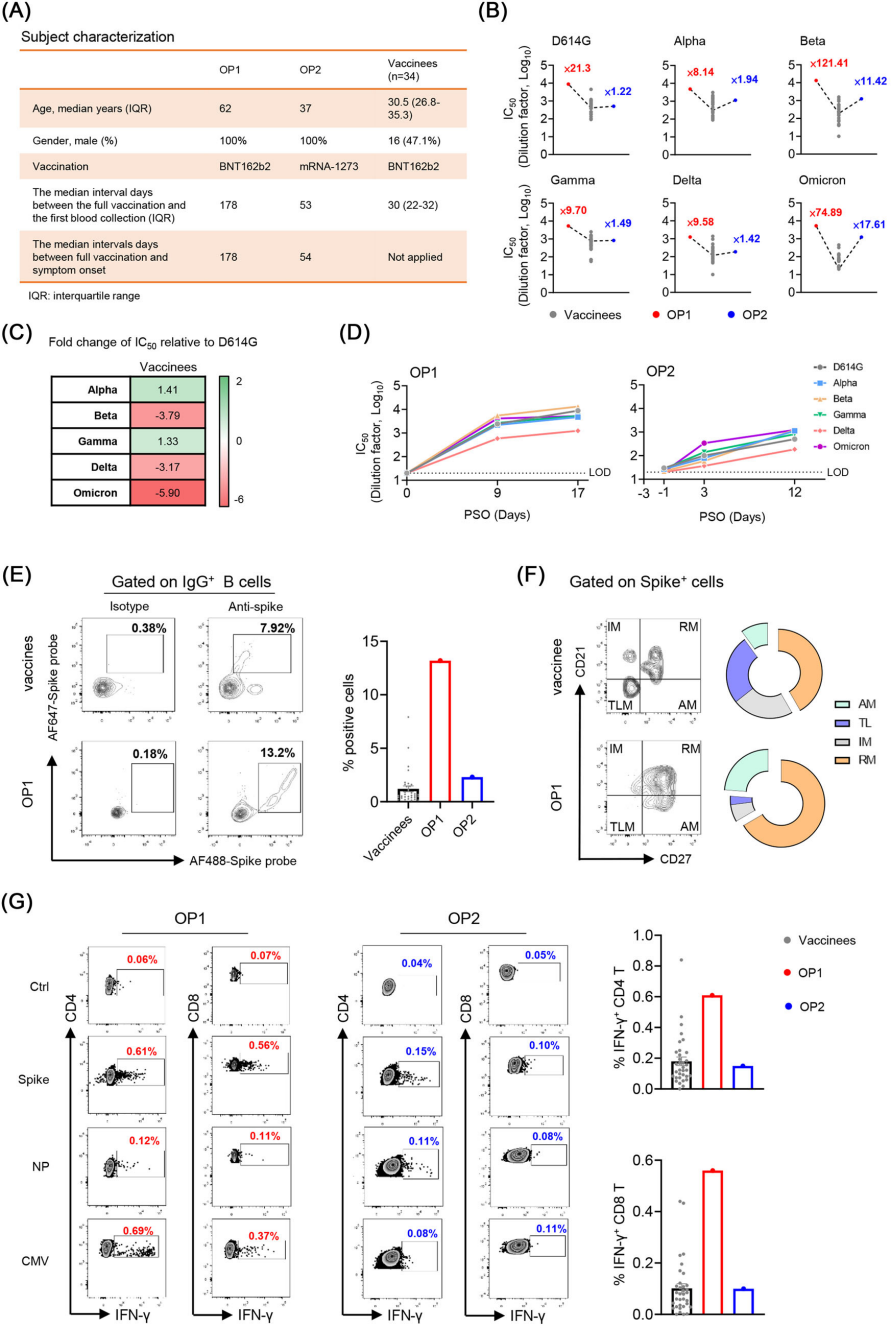
Cross‐reactive immune responses elicited by vaccine‐breakthrough infection of the SARS‐CoV‐2 omicron variant. (A) Characteristics of two omicron patients and 34 BNT162b2 vaccinees. (B) Neutralising antibody titres among the BNT162b2 vaccinees (grey) (*n* = 34) and two omicron patients (OP1: red and OP2: blue) at the peak response time. Neutralising antibody titres represent serum dilution required to achieve 50% virus neutralisation (IC_50_). The numbers indicate the fold of enhancement of IC_50_ values relative to mean titre measured among BNT162b2 vaccinees. (C) Fold‐change of mean IC_50_ values relative to the SARS‐CoV‐2 D614 G strain among the BNT162b2 vaccinees. (D) Longitudinal neutralising antibody titres (IC_50_) of OP1 and OP2 against the full panel of variants of concern (VOCs). Each symbol with colour coding represents an individual VOC. Dash line indicates the limit of detection (LOD) (1:20). (E–G) PBMCs were isolated from the blood collected at 11 and 12 days PSO of OP1 and OP2, respectively. PBMCs were further subjected to the measurement of antigen‐specific B‐ and T‐cell responses. (E) The gating strategy for SARS‐CoV‐2 Spike‐specific B cells by flow cytometry. AF488 and AF647 double‐positive cells were defined as Spike‐specific cells. Representative plots (left) and quantified results (right) are shown. (F) Phenotypes of Spike‐specific B cells were defined by using CD21 and CD27 markers (left). Pie chart shows the proportion of activated (AM), tissue‐like memory (TLM), intermediate memory (IM) and resting‐memory (RM) B cells. (G) PBMCs were subjected to the ICS assay against Spike or NP or CMV peptide pools. IFN‐γ^+^ cells were gated on CD4 and CD8 T cells, respectively (left). Quantified results (right) depict the percentage of IFN‐γ^+^ cells

We first measured the neutralising antibody titre (IC_50_) in their serum samples against the current panel of SARS‐CoV‐2 VOC pseudoviruses, including alpha (B.1.1.7), beta (B.1.351), gamma (P1), delta (B.1.617.2) and omicron (B.1.1.529) as compared with D614G (WT) (Figure 1B). We compared IC_50_ values of 34 local vaccinees, whose blood samples were collected around the mean 30 days after the second BNT162b2‐vaccination (Pfizer–BioNTech) (Figure 1A).^5^ Consistent with recent preprint publications by others, we found that the omicron variant showed the greatest resistance to BNT162b2 vaccine‐induced neutralisation with an average 5.9‐fold deficit relative to D614G (Figure 1C). Strikingly, however, the breakthrough infection was able to elicit cross‐reactive broadly neutralising antibodies (bNAbs) from the unmeasurable level (<1:20) to the mean IC_50_ value of 1:2929 (range 588.5–5508) at 9 days post symptoms onset (PSO) in OP1 and from the mean IC_50_ value of 1:24.3 to 1:854.5 at 12 days PSO in OP2, respectively (Figure 1D). Moreover, the amounts of NAbs in OP1 and OP2 were consistently higher than the mean IC_50_ values of BNT162b2 vaccinees across all VOCs tested (Figure 1B). In particular, there were 121.41‐ and 74.89‐fold higher IC_50_ values against beta and omicron in OP1 than those in BNT162b2 vaccinees (Figure 1B). Besides NAbs against the current panel of VOCs, OP1 also displayed enhanced IC_50_ values of NAbs against 15/16 SARS‐CoV‐2 variants with individual mutations or deletions, including the E484K mutation, which conferred significant resistance to vaccine‐induced NAbs (Figure S1). These results demonstrated that although the omicron VOC evaded BNT162b2 vaccine‐induced NAbs, the breakthrough infection could recall cross‐reactive bNAbs generally against all current VOCs in both OP1 and OP2.

Multicolour flow cytometry data showed no sign of severe immune suppression in OP1 and OP2 who had normal frequencies of T lymphocyte (no lymphocytopenia), stable conventional dendritic cell (cDC): plasmacytoid dendritic cell (pDC) ratio and normal myeloid‐derived suppressor cells (MDSCs) similar to mild and healthy subjects, as we described previously (Figure S2).^6^ We also measured the frequency of Spike‐specific IgG^+^ B cells, 13.2% in OP1 and 2.31% in OP2 were relatively higher than the mean 1.12% (range 0.004%–7.92%) found among BNT162b2 vaccinees around their peak responses (Figure 1E). Unlike SARS‐CoV‐2 infection in unvaccinated patients, who display predominantly tissue‐like memory (TLM) B‐cell response,^7^ Spike‐specific IgG^+^ B cells from OP1 and OP2 exhibited the dominant phenotype of resting memory (RM) (Figure 1F), which was commonly found among healthy BNT162b2 vaccinees.

We further measured their cross‐reactive T‐cell responses to the Spike and nucleocapsid (NP) peptide pools derived from the SARS‐CoV‐2 wildtype as compared with BNT162b2 vaccinees by intracellular cytokine staining (ICS). The cytomegalovirus (CMV) pp65 peptide pool was used as a positive control. We found that Spike‐ and NP‐specific CD4 IFN‐γ responses were 0.61% and 0.12% in OP1 and 0.15% and 0.10% in OP2, respectively (Figure 1G). Spike‐ and NP‐specific CD8 IFN‐γ responses were 0.56% and 0.11% in OP1 and 0.10% and 0.08% in OP2, respectively. Moreover, the Spike‐specific CD4 and CD8 T‐cell responses were relatively higher in OP1 or comparable in OP2 as compared with mean values in BNT162b2 vaccinees (CD4 T: mean 0.19% and CD8 T: mean 0.10%). As much weaker or unmeasurable T‐cell responses were found in severe COVID‐19 patients around the same period PSO,^6^, ^8^ T‐cell responses in OP1 and OP2 probably also contributed to disease progression control.

As the omicron variant caused a higher rate of vaccine‐breakthrough infection and reinfection than the delta variant,^9^ it is worrisome if such infections would lead to more severe sickness or death due to immune escape. In this study, we demonstrated that the omicron breakthrough infection rapidly recalled vaccine‐induced memory bNAbs and T‐cell immune responses, which very likely contributed to protection in OP1 and OP2. Our finding provides a probable immune mechanism underlying a recent report that most omicron patients had no signs of severe COVID‐19 as compared with the delta variant.^9^ Our findings, therefore, re‐emphasise the importance of complete vaccination coverage among human populations, especially in developing countries. Notably, the ongoing adaptive evolution of SARS‐CoV‐2 created an unprecedented demand of vaccines against VOCs.^10^ As similarly high amounts of bNAbs against both omicron and other VOCs were detected in OP1 and OP2, the rapid development of omicron‐based vaccine is a reasonable strategy as a booster vaccine to elicit and sustain long‐term cross‐protective immunity against COVID‐19.

## CONFLICT OF INTEREST

The authors declare that there is no conflict of interest.

## Supporting information

Supporting Information

Figure S1

Figure S2
