# Supplementary material for: Vaccine‐breakthrough infection by the SARS‐CoV‐2 omicron variant elicits broadly cross‐reactive immune responses
Source: Clin Transl Med. 2022 Jan 26;12(1):e720. doi: 10.1002/ctm2.720 (PMC8792449; doi:10.1002/ctm2.720)
Supplement: Supplementary file 1 — Supporting Information [file CTM2-12-e720-s003.docx]

**EXPERIMENTAL MODELS AND SUBJECT DETAILS**

**Human subjects**

This study was approved by the Institutional Review Board of the University of Hong Kong/Hospital Authority Hong Kong West Cluster (Ref No. UW 21-452). Written informed consent was obtained from all study subjects. Peripheral blood mononuclear cells (PBMCs) from healthy donors and patients were isolated from fresh blood samples using Ficoll-Paque density gradient centrifugation in our BSL-3 laboratory at the same day of blood collection. The majority of purified PBMCs were used for immune cell phenotyping whereas plasma samples were subjected to antibody testing. The rest of the cells were cryopreserved in freezing medium ([Synth-a-Freeze Cryopreservation Medium](https://www.thermofisher.com/order/catalog/product/A1254201), ThermoFisher Scientific) at 5 × 10^6^ cells/mL at −150°C.

**Pseudotyped viral neutralization assay**

To determine the neutralizing activity of subject’ plasma, plasma was inactivated at 56°C for 30 min prior to a pseudotyped viral entry assay. In brief, different SARS-CoV-2 pseudotyped viruses were generated through co-transfection of 293T cells with 2 plasmids, pSARS-CoV-2 S and pNL4-3Luc_Env_Vpr, carrying the optimized SARS-CoV-2 S gene and a human immunodeficiency virus type 1 backbone, respectively. At 48 h post-transfection, viral supernatant was collected and frozen at −150°C. Serially diluted plasma samples (from 1:20 to 1:14580) were incubated with 200 TCID_50_ of pseudovirus at 37°C for 1 h. The plasma-virus mixtures were then added into pre-seeded HEK293T-hACE2 cells. After 48 h, infected cells were lysed, and luciferase activity was measured using Luciferase Assay System kits (Promega) in a Victor3-1420 Multilabel Counter (PerkinElmer). The 50% inhibitory concentrations (IC_50_) of each plasma specimen were calculated to reflect anti-SARS-CoV-2 potency.

**Flow cytometry analysis**

For immune cell profile analysis, PBMCs were incubated for 10 min with Fc Block (BD Biosciences) in staining buffer (PBS containing 2% FBS) followed by staining with the indicated antibodies for 30 min at 4°C. For T cell responses, PBMCs were stimulated with 2 μg/mL COVID-19 Spike or NP peptide pool (15-mer overlapping by 11) or CMV pp65 peptide pool in the presence of 0.5 μg/mL anti-CD28 and anti-CD49d mAbs (BD Bioscience). Cells were incubated at 37°C overnight and BFA was added at 2 h post incubation. After overnight incubation, cells were washed with staining buffer (PBS containing 2% FBS) and stained with mAbs against surface markers. For intracellular staining, cells were fixed and permeabilized with BD Cytofix/Cytoperm (BD Biosciences) prior to staining with the mAbs against cytokines with Pern/Wash buffer (BD Biosciences). Stained cells were acquired by FACSAriaIII Flow Cytometer (BD Biosciences) inside a BSL-3 laboratory and analyzed with FlowJo software (v10.6) (BD Bioscience).
