# Supplementary figures and images for: Vaccine‐breakthrough infection by the SARS‐CoV‐2 omicron variant elicits broadly cross‐reactive immune responses
Source: Clin Transl Med. 2022 Jan 26;12(1):e720. doi: 10.1002/ctm2.720 (PMC8792449; doi:10.1002/ctm2.720)

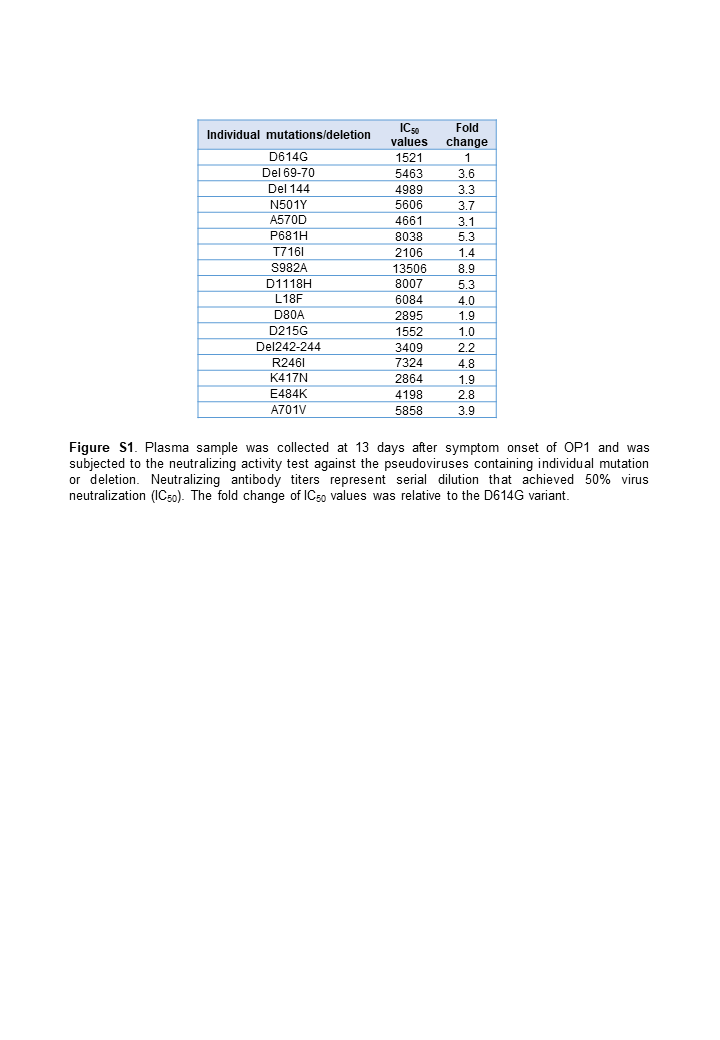

Supplement: Supplementary file 2 — Figure S1 [file CTM2-12-e720-s001.TIF]

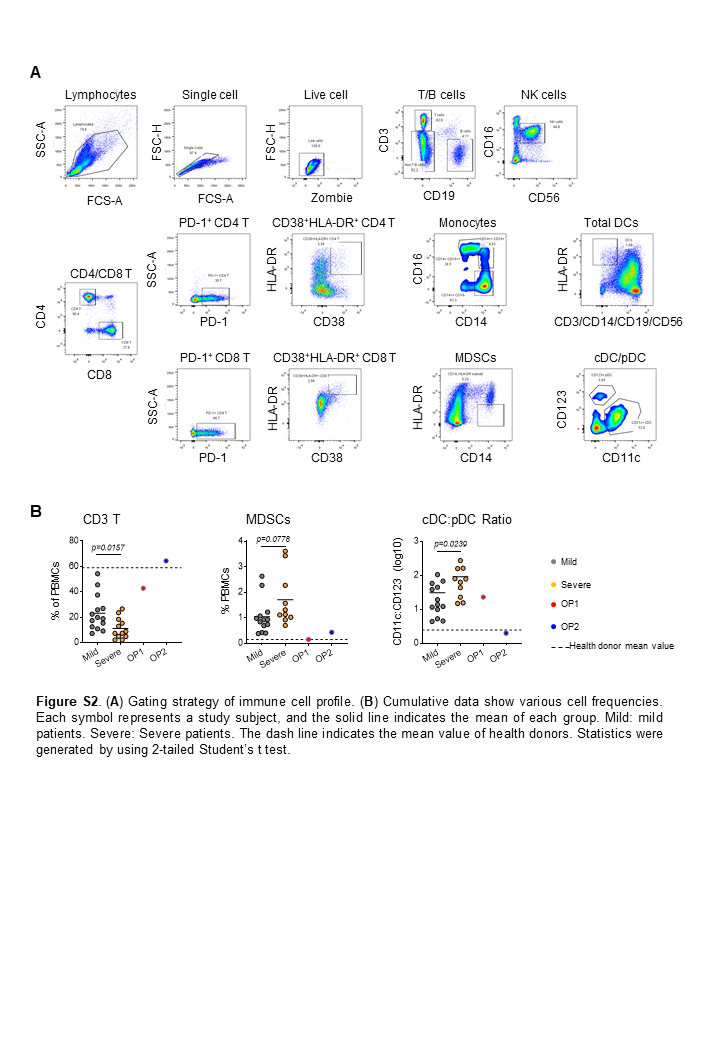

Supplement: Supplementary file 3 — Figure S2 [file CTM2-12-e720-s002.TIF]
